# The contribution of a negative colorectal screening test result to symptom appraisal and help‐seeking behaviour among patients subsequently diagnosed with an interval colorectal cancer

**DOI:** 10.1111/hex.12672

**Published:** 2018-02-19

**Authors:** Karen N Barnett, David Weller, Steve Smith, Robert JC Steele, Peter Vedsted, Sheina Orbell, Sue M Moss, Jane W Melia, Julietta Patnick, Christine Campbell

**Affiliations:** ^1^ University of Edinburgh Edinburgh UK; ^2^ Midlands and NW Bowel Cancer Screening Programme Hub Rugby UK; ^3^ University of Dundee Dundee UK; ^4^ Aarhus University Aarhus Denmark; ^5^ University of Essex Colchester UK; ^6^ Queen Mary University of London London UK; ^7^ University of Cambridge Cambridge UK; ^8^ University of Oxford Oxford UK

**Keywords:** colorectal cancer screening, interval cancer, negative screening result, symptom appraisal, help‐seeking, understanding of screening

## Abstract

**Background:**

Colorectal cancer (CRC) screening programmes using a guaiac faecal occult blood test (gFOBt) reduce CRC mortality. Interval cancers are diagnosed between screening rounds: reassurance from a negative gFOBt has the potential to influence the pathway to diagnosis of an interval colorectal cancer.

**Methods:**

Twenty‐six semi‐structured face‐to‐face interviews were carried out in Scotland and England, with individuals diagnosed with an interval colorectal cancer following a negative gFOBt result.

**Results:**

Participants reported they were reassured by a negative gFOBt, interpreting their result as an “all clear”. Therefore, most did not suspect cancer as a possible cause of symptoms and many did not recall their screening result during symptom appraisal. Among those who did consider cancer, and did think about their screening test result, reassurance from a negative gFOBt led some to “downplay” the seriousness of their symptoms with some interviewees explicitly stating that their negative test result contributed to a delayed decision to seek help.

**Conclusion:**

Screening participants need to be informed of the limitations of screening and the ongoing risk of developing colorectal cancer even when in receipt of a negative result: the importance of minimizing delay in seeking medical advice for colorectal symptoms should be emphasized.

## 1 INTRODUCTION

In the UK and other high‐income countries, colorectal cancer (CRC) is a leading cause of cancer‐related mortality.^1^, ^2^ Population‐based CRC screening programmes have been introduced in a number of countries and have been shown to reduce CRC mortality.^3^ Since 2006, the UK has introduced CRC screening programmes based on the guaiac faecal occult blood test (gFOBt), with colonoscopy offered to those who receive a positive result. UK programmes are currently switching to use of faecal immunochemical testing (FIT). Following a successful trial,^4^ the UK is also currently implementing national flexible sigmoidoscopy screening programmes that will run alongside existing biennial screening.

Despite CRC screening programmes being in place, uptake of CRC screening in the UK is approximately 55% and the majority of CRC cases will present symptomatically.^5^, ^6^, ^7^, ^8^, ^9^ Furthermore, gFOBt screening is associated with a high proportion of interval cancers: between 30% and 50% of all CRCs detected in the screened population in the Scottish and English pilot programmes.^10^, ^11^ Interval cancers include cancers that have developed between screening rounds and “missed” cancers following a false‐negative screening result; they have poorer survival when compared to screen‐detected CRC.^12^, ^13^, ^14^

A number of studies report that people who experience potential cancer symptoms rarely initially interpret them as such, often normalizing symptoms or attributing them to something else.^2^ The Model of Pathways to Treatment is a conceptual framework developed to describe the complexity of pathways leading to a cancer diagnosis and defines the processes within 4 key intervals (appraisal, help‐seeking, diagnostic and pre‐treatment) comprising key events from the detection of a bodily change through to the start of treatment.^15^, ^16^ The patient interval encompasses appraisal and help‐seeking.^17^ Evidence suggests that one of the main factors contributing to a long patient interval is non‐recognition of symptom seriousness resulting in increased time to presentation and diagnostic delay.^18^, ^19^

Previous reassurance from a healthcare provider for a similar symptom or receipt of a previous “all clear” diagnosis has been associated with delays in help‐seeking for potential cancer symptoms.^20^, ^21^ We and others have reported that participation in the NHS bowel screening programmes has the potential to lead to a delay in help‐seeking for some patients following the onset of symptoms, through over‐reassurance from a negative gFOBt result.^22^, ^23^ Unintended consequences, including over‐reassurance, from a negative or “normal” result have been demonstrated in breast cancer and other screening programmes.^24^, ^25^, ^26^

The aim of this study was to explore, through individual interviews with screening participants who were diagnosed with an interval colorectal cancer, if receiving a negative screening result in a CRC screening programme contributed to their subsequent response to symptoms potentially indicative of cancer, and their decision to seek medical advice.

## 2 METHODS

### 2.1 Design

Semi‐structured individual face‐to‐face interviews were carried out with patients diagnosed with an interval colorectal cancer following a negative gFOBt result through colorectal cancer screening (bowel screening programmes). Interviews were carried out within 6 months of patients receiving their cancer diagnosis.

### 2.2 Setting and recruitment

All patients newly diagnosed with a primary colorectal cancer and who had received a negative gFOBt result (within the preceding two years) from the Scottish bowel screening programme or the Midlands and North West screening programme hub were eligible to take part in the study. Eligible screening age for the two respective programmes was 50–74 years (Scotland) and 60–74 years (England). This study selected patients using a purposive approach, recruiting patients via the hospital clinic where they received or were currently receiving their cancer treatment in NHS Tayside, Scotland, and University Hospitals Coventry & Warwickshire NHS Trust, England. Lists of patients, reviewed by the multidisciplinary teams (MDT) following a colorectal cancer diagnosis, were sent quarterly to the local screening hub in Dundee, Tayside or Rugby, North West Midlands. Confirmation of receipt of a negative gFOBt result was confirmed through checking screening hub records, and the patient list was returned to the colorectal surgeon responsible for the patient's care.

Patient invitation letters were sent directly to the patient from the secondary care team on behalf of the research team, including a participant information booklet, response form and prepaid addressed return envelope. Once a response form was received by the researcher (KB), individuals were contacted by telephone to arrange a suitable time and date for the interview.

### 2.3 Exclusion criteria

Suitability based on clinical grounds (physical or mental health) was at the discretion of the secondary care team and primarily the patient's colorectal surgeon in charge of their care.

### 2.4 Data collection

Interviews were carried out by a female researcher, KB (MA, MSc, PhD), with previous training and experience in conducting qualitative research. Interviews took place between August 2013 and June 2014. Interviews lasted between 45 and 60 minutes, and signed consent was obtained before the interview commenced. All interviews were audio‐recorded with the participant's permission, professionally transcribed verbatim and anonymized. Field notes were made both during and after each interview. Audio files were deleted once the written transcripts had been received and verified by the researcher. Interviews took place in the patient's home, and some patients were accompanied by their spouse or friend during the interview.

### 2.5 Topic guide

Interviews explored two key domains: (i) the participant's “pathway to diagnosis” including: initial symptom appraisal, symptom progression, first presentation to any healthcare provider and any perceived delays, and (ii) the contribution, if any, of their negative gFOBt result to their symptom appraisal or decision to seek help.

### 2.6 Ethical review

The project was granted ethical approval from the South East Scotland Research Ethics Committee 01 (11/SS/0006).

### 2.7 Analysis

All transcripts were read by both the project researcher (KB) and the principal investigator (CC). Emerging themes were discussed and a coding frame agreed with additional codes included when appropriate as data collection progressed. Final themes were agreed through an iterative process involving the core and wider research group. Thematic analysis of the data was undertaken using NVivo software (QSR International, V.10) and was ongoing throughout the study to allow emerging themes to be fed back into the data collection. An inductive reasoning approach was adopted where themes (or categories) were identified through careful examination and comparison of the data which involved 6 stages: familiarization of the data, generating initial codes, searching for themes, reviewing themes, defining and naming themes and producing a report.^27^

## 3 RESULTS

### 3.1 Recruitment

A total of 27 participants were recruited, with 17 interviews carried out in Tayside, Scotland, and 10 in the Midlands and North West, England. Recruitment was ongoing throughout the study; 50% of patients who received an invitation to take part in the study agreed to be interviewed. Twenty‐one participants presented to their GP with symptoms prior to their diagnosis, two attended their GP for a routine diabetic health check, one participant was diagnosed following an emergency admission to hospital and two participants were diagnosed following a surveillance colonoscopy. One participant, wrongly identified, had received a positive gFOBt result in the screening programme, which led to their diagnosis: this interview was not included in the reported findings. A summary of study recruitment and participant demographics is provided in Table 1.

**Table 1:** Recruitment summary—interviews with interval bowel cancer patients in Tayside, Scotland, and North West Midlands, England

Interviews (N)	Location	Response rate (%)	Male (%)	Age range	Route to diagnosis (N)
17	Tayside, Scotland	17/33 (51)	9 (53)	52‐77	GP (15) Emergency Admission (1) Surveillance Colonoscopy (1)
10 (9^a^)	North West Midlands, England	10/21 (45)	6 (60)	63‐75	GP (8) Surveillance Colonoscopy (1) Positive gFOBt (1)^a^

a One patient transcript was not included in subsequent analysis due to a recruitment error—diagnosed following a positive guaiac faecal occult blood test (gFOBt).

### 3.2 Reported symptoms

Participants described a range of symptoms and symptom characteristics, which differed in frequency, duration and severity prior to receiving their colorectal cancer diagnosis. Reported symptoms included: noticeable blood on toilet paper or in the toilet bowl or stool following a bowel movement, constipation and/or diarrhoea, change in bowel habit, weight‐loss, indigestion, nausea and sickness, fatigue and pain. Participants who were diagnosed following a routine diabetic health check reported that they did not experience any symptoms preceding their cancer diagnosis.

### 3.3 Main themes

**Figure 1:**
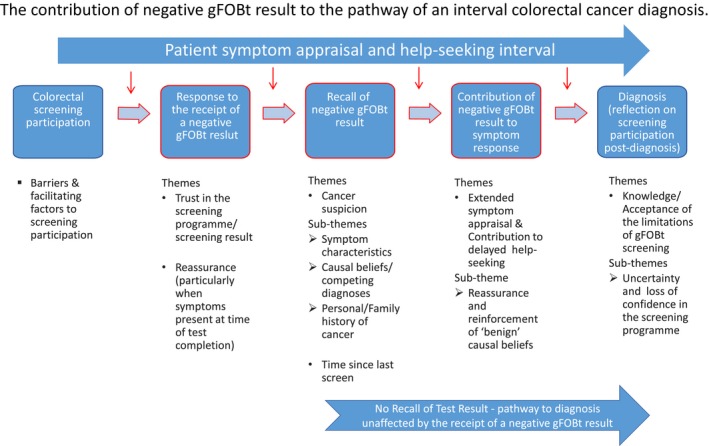
The contribution of a negative guaiac faecal occult blood test (gFOBt) result—identified themes and subthemes from patient accounts of their pathway to diagnosis of an interval colorectal cancer

We found the relationship between a negative gFOB result and a patient's diagnostic journey to be multifaceted, with varying opportunities or scenarios in which screening participation and the receipt of a negative result had the potential to influence the appraisal interval following the onset of symptoms (Figure 1). We identified three key stages where a negative gFOBt result had the potential to contribute to the appraisal and/or help‐seeking interval outlined in the Model of Pathways to Treatment,^15^ and a fourth stage where participants reflected on their screening participation following their interval colorectal cancer diagnosis. The potential impact of a negative gFOBt result at each stage was influenced by a number of emergent and cross‐cutting themes: (i) receipt of a negative screening (gFOBt) result *(Trust and Reassurance),* (ii) recall of a negative test result following the onset of symptoms *(Cancer Suspicion and Time Since Last Screen)*, (iii) the subsequent contribution, if any, of a negative gFOBt result to symptom response *(Extended Symptom Appraisal & Delayed Help‐seeking)* and (iv) reflection on the bowel screening programme following an interval colorectal cancer diagnosis *(Knowledge/Acceptance of the Limitations of gFOBt screening)*. These 4 key stages including the emergent themes and subthemes are shown in Figure 1.

### 3.4 Response to receipt of a negative gFOBt result

#### 3.4.1 Trust and Reassurance

Screening participation was associated with low levels of apprehension, with the receipt of a negative test result leading to positive emotions such as pleasure and relief. For some participants, who felt well and perceived themselves to be healthy, a negative result was simply confirmation of their health status. Participants portrayed a high degree of confidence and trust in their negative screening result describing it as “an all clear” that they “were ok” or that they had “passed.” Participants did not question the reliability of the test result when they received their result letter although some participants, following more in‐depth reflection, acknowledged the information or disclaimer, provided with the screening materials promoting symptom vigilance and help‐seeking between screening episodes.

I just thought that's good, I've got the all clear, I wasn't worried between sending it off and waiting on the letter to come back, I never actually gave it too much thought but obviously it's good to get the all clear. (Male, Tayside #02)

… they say it's fine so you take their word for it, they've done the tests… that's it until the next time it comes round and you just carry on. (Female, Tayside #01)

I did trust the thing, you know, the sample [gFOBt result], because I did, I'd sent them back, you know, and I always got a letter come back to say they were negative. (Female, Midlands #02)

Most participants did not recall experiencing symptoms when they completed their gFOBt. For a proportion of patients, the presence of symptoms contributed to their decision to participate. Among those who reported that they had symptoms when completing the test, or had been experiencing symptoms intermittently, a negative gFOBt result provided some degree of reassurance with regard to the seriousness or urgency of their symptoms.

I guess I had some reassurance from the fact that the screening tests had proved negative… I guess it made me feel that, okay, there is nothing drastically wrong at the minute. So I needn't, I needn't worry myself about it. Without fully appreciating, I suppose, what the screening test can and can't do. (Male, Midlands #03)

I took comfort each time [when he received a negative result]. I thought, there's nothing there so it can't be [cancer]. It must be alright… so it was a false reassurance, but as I say, it does say clearly on the letter, if you have any of these other symptoms, erm, go and see your doctor. (Male, Tayside #11)

### 3.5 Recall of negative gFOBt result following the onset of symptoms

#### 3.5.1 Cancer suspicion

We found that recall of participation in the colorectal screening programme, during symptom appraisal, was a key determinant in ascertaining whether receipt of a negative gFOBt result contributed to the patient interval. Among the interviewees, recall of their negative screening result was largely dependent on whether or not cancer was considered, even briefly, as a potential cause of their symptoms. Cancer suspicion and the recall and consideration of their gFOBt result were further influenced by a number of emergent subthemes including the characteristics of their experienced symptoms, competing diagnoses (or causal beliefs) and the presence of either a personal or family history of cancer.

Many participants reported that they did not recall, or think about, their negative screening result during the symptom appraisal interval and did not consider cancer as a potential cause of their symptoms. This was particularly evident among participants who described symptoms that were not readily associated with the bowel, for example feeling tired, acid reflux or hip pain, or when the cause of symptoms was assumed to be attributable to a benign cause (or a competing diagnosis), for example haemorrhoids or a stomach bug. Often during initial symptom appraisal, patients “normalized” or justified their symptoms, ascribing them to ageing or simply “overdoing” it.

We'd had bowel screening tests, or I had, the year before… I think it was, I can't remember offhand. And it was clear, so you sort of put that out of your mind, you know, out of the way. And I'd no change in my bowel habits… I think if my bowel movements had been erratic, or something, then I might have connected it somehow, I don't know. But it was so far over here [the pain], you know, I just thought it was my hip. (Female, Midlands #09)

Well, I didn't know, I never even thought of cancer, it just didn't enter my head; I just thought maybe I'd got internal piles or, you know, haemorrhoids, um, or, um, a, a fissure that was deep in, you know, inside; and never, ever gave it a thought that it could be a cancer, not at all. (Female, Midlands #01)

I put a lot of my problems down to tiredness, to age‐related as well. Simple as that, yeah. (Male, Midlands #10)

However, a smaller group of interviewees explicitly remembered considering their previous negative gFOBt result following the onset of their symptoms. These patients suggested that they suspected, or at least considered, colorectal cancer as a possible cause of their symptoms and/or were experiencing bowel‐associated symptoms either intermittently or at the time that they carried out their screening test. When symptoms persisted, increased in severity or additional symptoms developed, these participants applied a more serious illness label and/or suspected cancer as a possible cause of their symptoms. Similarly, participants with a personal or family history of cancer were more likely to attribute their symptoms to a potential cancer diagnosis.

I just put it down to constipation, like, you know? I wasn't getting heavy bleeding or anything like that, you know? So, It was only when I started getting these pains in the stomach that I started to think, well maybe this could be something worse… (Male, Tayside #08)

I had a family history of bowel cancer on my father's side and in the back of my mind right from the start it just… I thought, you know… this was the same symptoms that he had had… (Female, Tayside #05)

#### 3.5.2 Time since last screen

There was some evidence to suggest that patients who had *recently* participated in colorectal screening, prior to the onset of symptoms, for example in the preceding few months, were more likely to reflect on their negative screening result and recall the information provided with the result letter.

Every two years you have, like, a little pack come through the post and you're tested for that. And I'd not long completed one, so I assumed that it was nothing like that. I mean, the results had come back clear… (Male, Midlands #08)

I think because eh possibly [the] best part of two years had passed and [I] hadn't really read the literature, the information more recent, that I was [wasn't] aware of what was going on, if it happened just after I'd done the bowel screening a short period of time possibly after I might have been more aware but the information hadn't probably registered and stuck. (Male, Tayside #02)

### 3.6 The subsequent contribution, if any, of a negative gFOBt result to symptom response

#### 3.6.1 Extended symptom appraisal and delayed help‐seeking

Among the interviewees who considered their gFOBt result during the appraisal interval, there was a clear indication that for some, receipt of a negative gFOBt result contributed to an extended symptom appraisal interval, which in turn led to a delayed decision to seek help. Recall of a negative screening result helped to reassure participants by reinforcing pre‐existing “benign” causal beliefs (subtheme) and/or reducing any cancer suspicion, believing that colorectal cancer would be an unlikely cause of their symptoms having recently received an “all clear” from the bowel screening programme.

… because of thinking it was just a fissure, um, I automatically thought, well, if there was anything wrong it would show up in the screening, so it must be just a fissure. (Female, Midlands #01)

I didn't think that it could go wrong, you know. I just… I was really stressed at the time that this all happened, because of my sister [also had cancer diagnosis] but I was stressed then, before it all started with me, you know, I was stressed out, I thought, oh, God, I'm just you know, really worried now, I hope nothing's going to happen to me. And I think to myself, no, it can't, because I've had the tests, you know. (Female, Midlands #02)

I did assume that it was something else to start with cos I didn't consider, obviously at the back of your mind it is there [the possibility of it being cancer] but you think well, you had the screening it will probably be something else. (Female, Tayside #04)

Some participants were more explicit in their accounts that the reassurance provided by their negative test result contributed to them “downplaying” the potential severity or urgency of their symptoms. However, there was no strong indication that receipt of a negative result was the primary (or sole) driver for delaying consulting with a GP. It was described more as a contributing factor to the appraisal and help‐seeking interval in which a number of other factors and competing demands, such as caring for a spouse or going on holiday, influenced the timing of their decision to seek help.

I was going away to America; we were having a family holiday. We went to America, Disney World, ten of us, for two weeks, and I thought, well, I'll go back and see the doctor after I come back. (Female, Tayside #13)

For others, who were already more reluctant to seek help despite suspecting that their symptoms could be attributable to something serious, receipt of a negative gFOBt result provided an excuse to continue to ignore symptoms or allowed participants to justify (to themselves) their decision to delay seeking help.

[reflecting on having done the screening result a few months before] so I thought ‘och no ken it will be something else’, so I suppose I left it for longer than I should have just partly because I was always running about, but then eventually I went to the GP… (Female, Tayside #04)

Thinking back there's always perhaps a bit of wishful thinking, oh the test's okay then I don't need to worry about it. (Male, Midlands #03)

I mean, the results had come back clear… the screening do warn you that, um… the screening test did warn you that if you get any of these other problems, you should go to your GP… and, I mean, I just allowed that to be another excuse not to go. (Male, Midlands #08)

In contrast, one participant who presented promptly to the GP following an episode of bleeding attributed their prompt response to recalling the information contained within the screening result letter regarding symptom vigilance and advice on when to consult with a GP.

Because, they said that in the pamphlet, like, any signs of bleeding and all that shouldn't be ignored, like, you know? And, that's the reason I went up, just to make sure that I was okay. (Male, Tayside #08)

It's important to note that although a small number of patients were influenced by their negative test result, most interviewees believed that they presented promptly to their GP following the onset of symptoms (generally within a number of days or weeks). Early recognition of symptoms was usually followed by a short period of self‐monitoring where participants adopted a “watch and wait” approach to see if symptoms resolved by themselves. Generally, when symptoms persisted or progressed in severity, or following a period of self‐treatment using over‐the‐counted medications, for example for suspected haemorrhoids or constipation, participants chose to consult with a GP or HCP.

I just thought I'll wait a few weeks and see if it's… see if it improves and I was using proprietary stuff, you know. Erm, that I bought out the chemist. (Male, Tayside #15)

The presence of pre‐existing conditions or comorbidities and their treatment (prescribed or over‐the‐counter medications) was associated with an extended appraisal and diagnostic interval. This was evident among participants independent to any reflection on previous screening participation during their symptom appraisal. The impact of comorbidity on the pathway to diagnosis was two‐fold: (i) some participants reported that they attributed their new symptoms to a pre‐existing diagnosis and therefore did not seek immediate advice or view the symptoms as serious, and (ii) once a decision had been made to seek help, referral to secondary care was sometimes delayed due to an initial focus, both by the patient and by the GP, on alternate treatments or investigations for a pre‐existing condition.

Well, I've got MS as well, you see so sometime I'll be thinking the headaches and that or the dizziness, ‘cause that's one of the things I had when I had the MS… oh I had a real dizzy spells, … so I was thinking it was that [laugh]. (Male, Tayside #12)

Yeah, I was thinking, oh, it's my Fibregel playing up and then we changed to the Movicol [history of bowel prolapse], I think I went two or three times [to see GP], because we tried… we tried Movicol and we tried, erm, something else with Senna… and we tried that for, like, a couple of weeks and it wasn't getting any better. (Female, Midlands #07)

### 3.7 Reflection on the bowel screening programme following an interval colorectal cancer diagnosis

#### 3.7.1 Knowledge/acceptance of the limitations of gFOBt screening

While some participants recalled their negative gFOBt result during the appraisal interval, others only considered their participation in the colorectal screening programme *after* they had received their cancer diagnosis. These participants tended to respond in one of two ways when reflecting on their negative gFOBt result, which was dependent on their knowledge and acceptance of the limitations associated with gFOBt screening. For some, the process of being given a perceived “all clear” followed by a colorectal cancer diagnosis led to some degree of uncertainty regarding the accuracy of their test result and a loss of confidence in the screening programme (subtheme). In contrast, others reported that they understood that the gFOB test was not 100% effective and was not a guarantee that they did not have or would not develop cancer in the future, recognizing the importance of monitoring symptoms and recalling the disclaimer that accompanies the screening result letter.

I will admit when I was told what I had, I thought oh “so much for your bloody test”, but I mean that was it, just left it at that… but it's like everything else erm what a terrible thing to say but don't put a great store in what these samples say… (Female, Tayside #01)

I mean the fact that I took, I… I had the sticks [gFOBt screening test], um, made me think to myself, you know, it did make… it has made me wonder now how good the sticks are? (Female, Tayside #13)

You know it comes down to personal responsibility, you get the negative result, you are told that if you have any symptoms, now I did have symptoms the bleeding but I chose to ignore it because I thought it was something else. (Female, Tayside #04)

Overall, interviewees remained very positive towards colorectal cancer screening and the associated benefits and showed a willingness for continued participation, including encouraging others to participate in the programmes.

Let's just say I'll still keep in with the screening programme but I won't have the same feeling of certainty about it. (Female, Tayside #03)

## 4 DISCUSSION

### 4.1 Principal findings

In this study, we examined whether the receipt of a negative gFOBt result contributed to the pathway to diagnosis among screening participants subsequently diagnosed with an interval colorectal cancer. Patient accounts highlighted the variability and complexity of presenting symptom type and contributing factors that influenced the patient interval. Participants placed a high level of trust in their screening gFOBt result, interpreting it as an “all clear” and seldom questioned the reliability of the result or the limitations of the screening test when they received their result letter. The majority of participants did not consider their previous gFOBt result (nor consider a potential colorectal cancer diagnosis) following the onset of symptoms, and therefore, reassurance from a negative gFOBt did not contribute to a delay in the patient interval. However, for a smaller group of patients who did consider colorectal cancer as a possible cause of their symptoms (or were experiencing symptoms intermittently or at the time they completed their screening test), a negative gFOBt result did influence the patient interval and contributed to a delay in seeking help. A negative gFOBt result led participants to “downplay” the seriousness of their symptoms by reducing their cancer suspicion and reinforcing “benign” causal beliefs having been reassured by a recent “all clear” test result. Pre‐existing conditions, particularly when the characteristics of any new symptoms were similar to those previously experienced, led to some delay across the symptom appraisal, help‐seeking and diagnostic interval.^15^

### 4.2 Comparison with existing literature

The processes within the appraisal interval as part of the Model of Pathways to Treatment,^15^ namely “patient appraisal and self‐management,” draw on a number of psychological theories including Leventhal's Common Sense Model of Illness self‐regulation and the Andersen Model of Total Patient Delay.^16^, ^28^ Consistent with our findings, Leventhal's model explains that initial symptom appraisal, or recognition of a bodily change, when symptoms do not exceed a certain level of interference, leads to symptoms being normalized or dismissed. However, when symptoms persisted, or increased in severity (eg exceeded a threshold of interference), patients adopted a different approach where they self‐monitored their symptoms or sought self‐treatments prior to making the decision to seek medical advice. Furthermore, as reported in the literature, we found that many participants did not attribute their symptoms to a potential cancer diagnosis despite some experiencing a colorectal cancer “alarm” symptom, for example blood in stool.^2^

Lack of recognition of the seriousness of a symptom has been reported extensively in the literature as one of the main contributing factors to a delayed patient interval.^18^, ^19^ In both the present study and the study carried out by Solbjor and colleagues examining interval breast cancers following mammography screening, a negative screening result was shown to impact on the patient interval primarily through reassurance which led some participants to “downplay” the seriousness of their symptoms and to assume a benign cause.^26^ Hall and colleagues found that a negative screening result, along with a number of other factors including absence of blood or pain, or “feeling well,” was viewed as reassuring.^23^ In the same way, reassurance from a previous “all clear” investigation has been associated with a delay in help‐seeking even when symptoms persisted or occurred a number of years later with some patients believing that they would not be taken seriously if they returned with similar symptoms.^20^, ^21^ The added complexity of the presence of comorbidities (or competing diagnoses) and the associated delay on the diagnostic pathway potentially resulting in late‐stage disease is an important and recognized challenge for healthcare providers,^29^, ^30^ particularly in the light of the recent evidence published by Torring and colleagues who report any delay to the primary care interval to be associated with more advanced colorectal cancer.^31^

Interval colorectal cancer has been shown to have an adverse effect on trust in FOBt screening, with the same study reporting poorer quality of life among interval cancer patients when compared to those with screen‐detected disease.^32^ Similarly, this has been demonstrated in mammography screening where a diagnosis of an interval breast cancer influenced patient's trust, although not to the point of creating distrust, where women instead saw themselves as exceptions in an otherwise beneficial screening programme.^33^ We also found that despite some interviewees suggesting a loss of confidence in the screening programme following their diagnosis, participants generally remained positive towards the overall benefits of screening and expressed a willingness for continued participation.

### 4.3 Strengths and limitations

Participants were recruited from hospitals in both Scotland and England, providing narratives from cancer patients who participated in different area‐based bowel screening programmes. Carrying out semi‐structured interviews within six months of diagnosis permitted an in‐depth exploration of the participant's pathway to diagnosis; nevertheless, accounts are retrospective and therefore subject to recall and framing bias. We did not have access to any clinical or demographic information for those who declined to be interviewed, nor the number that the hospitals did not consider eligible to contact: their experiences and perspectives may differ.

### 4.4 Implications for practice and policy

Although reassurance from a negative gFOBt result is appropriate for most screening participants, there is a risk of over‐reassurance for some. Methods of increasing understanding of the limitations of gFOBt screening (and indeed of FIT screening) and the ongoing risk of developing cancer despite receiving a negative test result are needed; the significance of any associated delay in seeking medical advice in terms of clinical outcomes also merits further exploration. Similarly, finding effective ways to engage with screening participants that complement existing initiatives in primary care with regard to symptom vigilance and prompt help‐seeking behaviour remains an important challenge; there is a need to develop nuanced health messages to inform screening participants of the importance of help‐seeking for vague as well as alarm symptoms following a negative test result, particularly in the presence of comorbidities. The use of FIT as a diagnostic tool in primary should allow more streamlined diagnostic pathways for all patients.^34^

## CONFLICT OF INTEREST

All authors declare that they have no competing interests.
